# Characterization of Vaginal Microbiota in Women With Recurrent Spontaneous Abortion That Can Be Modified by Drug Treatment

**DOI:** 10.3389/fcimb.2021.680643

**Published:** 2021-08-19

**Authors:** Fuju Zhao, Yisheng Chen, Jing Gao, Mengyin Wu, Cui Li, Zhiheng Wang, Nali Huang, Lefang Cui, Meirong Du, Chunmei Ying

**Affiliations:** ^1^Clinical Laboratory, Obstetrics and Gynecology Hospital of Fudan University, Shanghai, China; ^2^Clinical Laboratory, Huadong Hospital of Fudan University, Shanghai, China; ^3^Key Laboratory of Medical Molecular Virology of the Ministry of Education/Ministry of Health (MOE/MOH), Department of Medical Microbiology and Parasitology, School of Basic Medical Sciences, Fudan University, Shanghai, China; ^4^Sinotech Genomics Co., Ltd., Shanghai, China; ^5^NHC Key Laboratory of Reproduction Regulation (Shanghai Institute of Planned Parenthood Research), Fudan University Shanghai Medical College, Shanghai, China; ^6^Shanghai Key Laboratory of Female Reproductive Endocrine Related Diseases, Obstetrics and Gynecology Hospital of Fudan University, Shanghai, China

**Keywords:** recurrent spontaneous abortion, RSA, vaginal microbiome, aspirin, metformin, 16S rRNA gene

## Abstract

**Objective:**

The role of vaginal microbiota in recurrent spontaneous abortion (RSA) remains unknown. The purpose of this study was to investigate characteristics of vaginal microbiota and the effects of drug treatment on vaginal microbiota of patients with RSA.

**Methods:**

A case-control study was performed, in which non-pregnant patients who experienced RSA were selected and divided into untreated and drug-treated groups. Drug-treated patients were subdivided into the metformin group, metformin plus aspirin group, and other drugs group. Healthy women who had live births and never experienced spontaneous abortion were enrolled in the control group. Characteristics of vaginal microbiomes of patients with RSA and healthy women and the impact of drug treatment on the microbiome was evaluated *via* 16S rRNA gene sequencing of the V3-V4 region using the Illumina MiSeq platform.

**Results:**

Women who underwent RSA had lower microbial richness than healthy women. Compared to controls, the relative abundance of seven taxa (*Megasphaera*, *Sneathia sanguinegens, Pseudomonas*, *Sphingomonas, Rhodococcus, Burkholderia- Caballeronia*-*Paraburkholderia*, and *Corynebacterium*_*1*) in the patient’s vaginal microbiota changed significantly, which may be closely related to RSA. The composition of the vaginal microbial community in RSA patients was altered by drug treatment. Metformin combined with aspirin treatment significantly increased the relative abundance of vaginal *Lactobacillus* spp. in patients.

**Conclusion:**

An altered vaginal microbiome composition might be associated with RSA, which could be modified by drug treatment. The effect of metformin combined with aspirin on vaginal *Lactobacillus* is worthy of attention.

## Introduction

The American Society for Reproductive Medicine and the European Society of Human Reproduction and Embryology (ESHRE) defines recurrent spontaneous abortion (RSA) as two or more failed pregnancies. RSA affects approximately 1%–5% of couples trying to conceive (Practice Committee of the American Society for Reproductive Medicine, 2012; ESHRE Guideline Group on RPL et al., 2018). Existing research shows that the etiology of RSA includes genetic, anatomic, infective, thrombophilic, endocrine, and immune factors (Evaluation and treatment of recurrent pregnancy loss: a committee opinion, 2012; Rai and Regan, 2006; ESHRE Guideline Group on RPL et al., 2018; Khalife et al., 2019). Unfortunately, 50% patients do not have any of the above conditions and are hence considered idiopathic (Khalife et al., 2019; Green and O’Donoghue, 2019). This adds to patients’ financial and psychological burdens and also limits effective interventions and therapeutics. To date, international groups have not reached a consensus on the standard evaluation of RSA (Khalife et al., 2019). Various therapeutic strategies have been evaluated to decrease the rate of pregnancy loss. Low-dose aspirin, low-dose steroids, heparin, levothyroxine, progesterone, intralipid, and metformin are generally chosen to treat women with RSA through single or combined use (Rai and Regan, 2006; Vesce et al., 2014; Khalife et al., 2019; Green and O’Donoghue, 2019; Ou and Yu, 2019). The reported effects of the above-mentioned drug treatments are inconsistent. In-depth research on the etiology of RSA to determine the true causes will ensure a unified and standardized treatment in the future, thereby avoiding unreasonable treatment and reducing the financial and psychological burden of families with RSA.

With the development of high-throughput sequencing technology, an increasing number of microbiota inhabiting different parts of the human body have been associated with various diseases, which has expanded our understanding of the underlying mechanisms involved in these conditions. Although most of these studies focused on the gastrointestinal microbiome, the role of the vaginal microbiome for female reproductive health has attracted the attention of researchers over time (Chow and Mazmanian, 2010; Nikoopour and Singh, 2014; Younes et al., 2018). Two scientific conferences were held in Amsterdam to discuss the topic of women and vaginal microbiota in 2015 and 2016 to emphasize the particularity of the female microbiome and summarize the current research (Younes et al., 2018). Studies showed that the vaginal microbiota of healthy women are mainly colonized by *Lactobacillus* spp. including *L. crispatus*, *L. gasseri*, *L. iners*, and *L. jensenii* (Aroutcheva et al., 2001). Notably, some healthy women do not have *Lactobacillus* dominance, especially South African black women. The causative factors leading to ethnic and geographic differences in vaginal bacteria remain unclear (Anahtar et al., 2018). Ravel et al. analyzed the vaginal microbiota of women of childbearing age in North America and proposed five vaginal community structure types (CSTs) that have been commonly adopted in vaginal microbiome studies. The vaginal microbiota is affected by some physiological factors such as menarche, menstrual cycle, pregnancy, menopause, and other hormonal changes (Achilles et al., 2018; Greenbaum et al., 2019). Hickey et al. suggested that vaginal pH in healthy premenarche girls after menarche was usually higher than the level in healthy adult women, even with high proportions of *Lactobacillus* (Hickey et al., 2015). The overall diversity of the vaginal microbiome during pregnancy decreases while the stability increases. In addition, the abundance of the dominant *Lactobacillus* in the vagina increases significantly during pregnancy, which reduces the vaginal pH and strengthens the vagina’s ability to resist pathogenic microorganisms. After delivery, the microbiota revert to be similar to those in non-pregnant women (Neuman and Koren, 2017). Vaginal microbiota composition is also affected by exogenous factors including hygienic practices, contraceptive method, sexual behavior, stress, diet, exercise, drugs, and rectal colonization (Muzny and Schwebke, 2016; Abdelmaksoud et al., 2017; Achilles et al., 2018; Turpin et al., 2019; Song et al., 2020). According to existing literature reports, imbalanced vaginal microflora is related to the following diseases, including but not limited to, bacterial vaginosis, sexually transmitted infections, preterm birth (PTB), gynecological cancers, preterm pre-labor rupture of the fetal membranes, recurrent implantation failure, and polycystic ovary syndrome (PCOS) (Brotman, 2011; Muzny and Schwebke, 2016; Champer et al., 2017; Freitas et al., 2018; Brown et al., 2018; Elovitz et al., 2019; Fu et al., 2020; Hong et al., 2020). The relationship between PTB and the vaginal microbiome was recently described in several studies, with few reports on the vaginal microbiome of RSA (Zhang et al., 2019).

In the past, based on traditional serology and culture methods, *Mycoplasma hominis*, *Ureaplasma urealyticum*, *Listeria monocytogenes*, *Gardnerella vaginalis*, and other less frequent pathogens were identified more often in patients experiencing spontaneous abortion (Penta et al., 2003; Kuon et al., 2017). However, Contini et al. proposed that *Mycoplasma hominis* and *Ureaplasma urealyticum* may not cause embryo loss after analyzing bacterial DNA in the aborted tissues of women with early pregnancy loss and women underwent voluntary interruption of pregnancy *via* quantitative real-time PCR methods (Contini et al., 2018). With the development of technology, we will have a better understanding of the pathogenesis of abortion. Recent application of 16S rRNA gene-based metataxonomics for exploring the association between vaginal bacterial composition and abortion suggested that vaginal bacterial composition of abortion in the first trimester was related to decreased *Lactobacillus spp*. (Al-Memar et al., 2019). However, the authors could not conclusively show when the loss of *Lactobacillus* spp. occurs. In addition, a recent study has found that *Atopobium*, *Streptococcus*, and *Prevotella* were significantly more abundant in patients with unexplained recurrent miscarriage, while two (*Lactobacillus* and *Gardnerella*) taxa were overrepresented in controls (Zhang et al., 2019). As this study was limited by a small sample size, a definitive conclusion could not be drawn.

In our study, 108 patients with RSA were enrolled to characterize the vaginal microbiota composition in non-pregnant patients with RSA and assess the effects of drugs on bacterial composition using 16S rRNA gene-based metataxonomics.

## Materials and Methods

### Subject Recruitment and Ethical Approval

Non-pregnant patients in the Department of Reproductive Immunity, Obstetrics, and Gynecology Hospital of Fudan University who had experienced RSA were selected and divided into untreated and drug-treated groups. Drug-treated patients were subdivided into the metformin group, metformin plus aspirin group, and other drugs group. Healthy women who had live births and never experienced spontaneous abortion were enrolled in the control group. The exclusion criteria were as follows: women who were menstruating or had sexual intercourse in the last 72 h. Participants on any antibiotic treatment or who underwent vaginal lavage in the 2 weeks prior to swab collection were also excluded. All participants provided written informed consent and gave permission for collection of their vaginal specimens and related clinical information. The study was approved by the Ethics Committee of the Obstetrics and Gynecology Hospital of Fudan University.

### Sample Collection and Physiological and Biochemical Analyses

Vaginal specimens were collected from each participant from the lateral wall of the vagina using a sterile swab. One sample was used for physiological and biochemical testing immediately including hydrogen peroxide, pH, leukocyte esterase, and sialidase activity according to manufacturer’s instructions (Bioperfectus Technologies, Jiangsu, China). Other swabs were placed immediately in an ice box and stored at -80°C until further analysis. Women enrolled in this study were asked to fill out a questionnaire to collect additional demographic and medical information (age, body mass index [BMI], drug use, gynecological and obstetric history, exercise intensity, smoking, hygiene habits, and mental health conditions).

### DNA Extraction and 16S rRNA Sequencing

Total nucleic acid extracted from each vaginal swab was performed using the FastDNA^®^ SPIN Kit for Soil (MP Biomedicals, Ohio, USA) according to manufacturer’s instructions. DNA purity and concentration of each sample were measured with the NanoDrop2000 (Thermo Fisher Scientific, Wilmington, USA). The quality of extracted DNA was determined by 1% agarose gel electrophoresis. The V3/V4 regions of the 16S rRNA gene were amplified by the PCR system with primers 338F (5’-ACTCCTACGGGAGGCAGCAG-3’) and 806R (5’-GGACTACHVGGGTWTCTAAT-3’) to further verify the quality of the sample DNA. The PCR system was performed in triplicate with 20 μL mixtures containing 4 μL of 5 × FastPfu buffer, 2 μL of 2.5 mM deoxynucleoside triphosphates, 0.8 μL of each primer (5 μM), 0.4 μL of FastPfu polymerase, 10 ng of template DNA, and double-distilled water to make up the total volume. PCR reactions were performed on ABI GeneAmp^®^ 9700 (Thermo Fisher, Waltham, MA, USA) using the following cycling parameters: 95°C for 3 min, followed by 27 cycles at 95°C for 30 s, 55°C for 30 s, and 72°C for 45 s, with a final extension at 72°C for 10 min. The amplicons were extracted from a 2% agarose gel purified using the AxyPrep DNA Gel Extraction Kit (Axygen Biosciences, Union City, CA, USA) and quantified by QuantiFluor™-ST (Promega, Madison, WI, USA) according to the manufacturer’s instructions. Equimolar quantities of purified amplicons were pooled and paired-end sequenced (2 × 300) on the Illumina MiSeq PE300 (Illumina, San Diego, CA, USA) according to the manufacturer’s specifications for MiSeq Reagent Kit v3.

### Bioinformatics and Microbiota Analysis

Statistical analyses were performed using ‘R’ language and other packages. Raw read-pairs were merged with FLASH and quality filtered by Trimmomatic (Illumina). Sequence alignment and classification was performed using the Silva bacterial database (www.arb-silva.de/) and the RDP (Ribosomal Database Project) database reference sequence files (http://rdp.cme.msu.edu). The taxonomy of operational taxonomic units (OTUs) clustering at a 97% sequence identity threshold was analyzed by the RDP Classifier algorithm (https://sourceforge.net/projects/rdp-classifier/, version 2.11) at each classification level (kingdom, phylum, class, order, family, genus, species). The vaginal CSTs of each sample was categorized as follows. CSTI is dominated by *L.crispatus*, CSTII by *L. gasseri*, CSTIII by *L. iners*, and CSTV by *L.jensenii.* CSTIV had a diverse set of facultative and strict anaerobes.

Alpha-diversity was inferred by the Chao1 estimator and Shannon–Wiener index. Differences in community richness and diversity between sample groups were determined with nonparametric one-sided Wilcoxon rank-sum tests. PCoA was used to estimate differences in beta diversity. Differentiation of the overall microbial community structure of each group was assessed with nonparametric multivariate analyses of variance (MANOVA). The nonparametric factorial Kruskal-Wallis rank-sum test was used to identify taxa showing differentially abundant features between two groups. Linear discriminant analysis (LDA) was used to estimate the contribution of differentially abundant taxa to group differentiation with the LEfSe software. Fisher’s exact test was used to analyze whether there was a significant difference in the frequency of five CSTs between each group. Statistical analyses were performed using SPSSv.17.0 software (SPSS Inc., Chicago, IL, USA).

## Results

### Clinical Characteristics of the Participants and Sequencing Results

A total of 120 patients diagnosed with recurrent miscarriage and 20 healthy women were recruited and assigned to the case and control groups, respectively. We successfully analyzed 126 specimens with 16S rRNA gene sequencing, including 108 samples from the RSA group and 18 from the control group. The clinical and demographic information of participants are shown in **Table 1**. All participants in the control and case were non-smokers and non-vegetarians. Of the enrolled 108 cases, 65 (60.18%) were newly diagnosed patients not taking medication and 43 (39.82%) were under treatment including metformin (*n* = 9), metformin combined with aspirin (*n* = 9), and other drugs (*n*=25). Other than age and leukocyte esterase (LE) activity (patients vs. controls, p < 0.05), there were no significant differences in other parameters including BMI, hygiene, exercise intensity, self-rating depression scale (SDS), self-rating anxiety scale (SAS), H_2_O_2_, sialidase activity, and pH (all *p* > 0.05). The MANOVA was used to evaluate the influence of age on differences between groups, we did not detect significant differences (*p* = 0.396).

**Table 1:** Clinical and demographic characteristics of the study population.

	NM group	DT group	P value (NM *vs.* DT)	Control group	P value (NM *vs.* Control)	P value (DT *vs.* Control)
Total number	65	43		18		
Age (years, mean ± SD)	31.5 ± 4.2	30.2 ± 3.6		36.6 ± 3.6		
≤29	25(38.5)	20(46.5)	0.19	1(5.6)	0.00	0.00
30–35	28(43.1)	20(46.5)		4(22.2)		
≥36	12(18.4)	3(7)		13(72.2)		
BMI (kg/m^2^ mean ± SD)	22.4 ± 3.2	22.8 ± 3.4		22.5 ± 3.8		
Underweight (<18.5)	4(6.1)	2(4.7)	0.60	2(11.1)	0.86	0.62
Normal weight (18.5–24)	44(67.7)	28(65.1)		11(61.1)		
Overweight (>24)	17(26.2)	13(30.2)		5(27.8)		
Hygiene (frequency of washing the vulva)						
Once a day	54(83.1)	38(88.4)	0.45	14(77.8)	0.60	0.29
Clean once every 2-3 days	11(16.9)	5(11.6)		4(22.2)		
Exercise intensity						
Low	56(86.2)	36(83.7)	0.72	15(83.3)	0.76	0.97
Medium	9(13.8)	7(16.3)		3(6.7)		
SDS						
Positive	14(21.5)	7(16.3)	0.51	4(2.2)	0.95	0.58
Negative	51(78.5)	36(83.7)		14(7.8)		
SAS						
Positive	5(7.7)	2(4.7)	0.53	3(6.7)	0.25	0.12
Negative	60(92.3)	41(95.3)		15(3.3)		
RSA						
2	36(55.4)	24(55.8)	0.78			
3	21(32.3)	16(37.2)				
≥4	8(12.3)	3(7)				
H_2_O_2_						
Positive	64(98.5)	42(97.7)	1	18(100)	1	1
Negative	1(1.5)	1(2.3)		0(0)		
pH						
>4.5	10(15.4)	6(14.0)	1	2	1	1
≤ 4.5	55(84.6)	37(86.0)		16		
Sialidase activity						
Positive	0(0)	0(0)		0(0)		
Negative	65(100)	43(100)		18(100)		
Leukocyte esterase activity						
Positive	1(1.5)	2(4.7)	0.656	0(0)	0.001	0.001
Weakly positive	29(44.6)	20(46.5)		1(5.6)		
Negative	35(53.9)	21(48.8)		17(94.4)		

NM, no medication; DT, drug treatment; BMI, body mass index; SDS, self-rating depression scale; SAS, self-rating anxiety scale; RSA, recurrent spontaneous abortion.

After preprocessing, 5,758,981 reads were obtained from all samples, with an average read count of 45,706 reads per sample (range: 30,182–92,508). After clustering, the Shannon–Wiener curve of each sample was nearly a straight horizontal line, which demonstrated that the sequencing depth of each sample was sufficient (**Supplementary Figure S1**).

### The Diversity of Vaginal Microbiomes in RSA and Response to Drug Treatment

**Figure 1:**
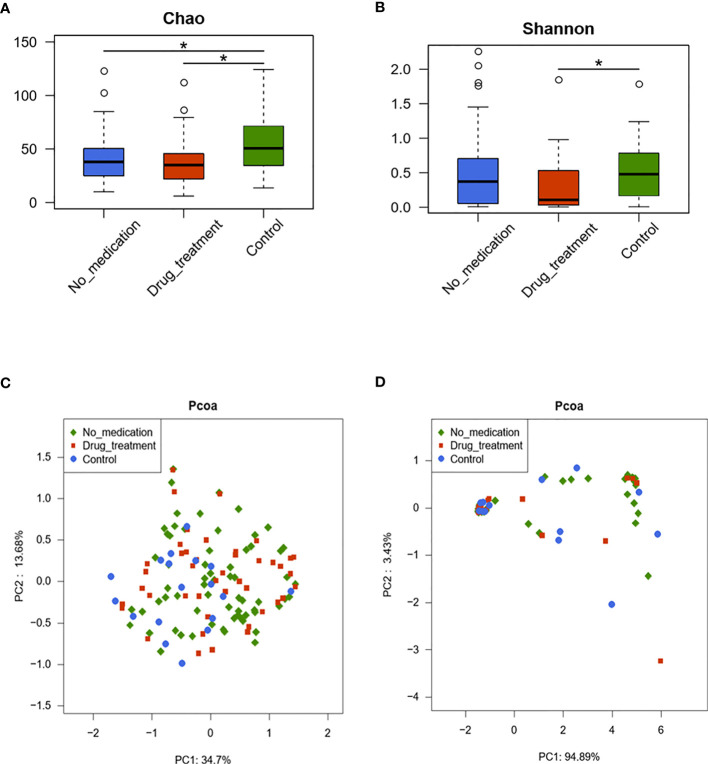
The diversities of vaginal microbiota in the three groups are shown by the Chao index **(A)**, Shannon–Wiener index **(B)**, and principal coordinate analysis (PCoA) plot created based on the unweighted **(C)** and weighted **(D)** UniFrac distances. The values show the percentages of total community variation explained. **(A, B)** *p*-values were calculated using the one-sided Wilcoxon rank-sum tests. *p < 0.05.

Individual comparison showed that community richness decreased in the no-medication and drug treatment groups compared to the control group (*p* < 0.05); meanwhile, there was no significant difference in richness between the drug-treated and no-medication groups (*p* = 0.67), indicating reduced richness in the cases (**Figure 1A**). Community diversity was calculated by the Shannon–Wiener index (0.49 ± 0.54 for the no-medication group, 0.36 ± 0.39 for the drug-treated group, and 0.56 ± 0.46 for the control group) and showed significant differences in the drug-treated and control groups (*p* = 0.01) but not between the no-medication and control groups (*p* = 0.24) (**Figure 1B** **)**. The Shannon–Wiener index values showed similar microbial diversity in the no-drug treatment cases and controls. However, the diversity of vaginal microflora decreased after drug treatment. These results revealed a decrease of RSA community richness and a similar diversity with that of healthy women, but this diversity would be reduced after drug treatment.

Two-dimensional principal coordinate analysis (PCoA) based on unweighted and weighted UniFrac distances was applied to illustrate characteristics of beta diversity between the case and control groups. **Figure 1C** shows the unweighted PCoA plot, which revealed clustering of the no-medication and drug-treated groups away from the control group. However, overall microbiota profiles clustered together based on analyzing with weighted UniFrac PCoA (**Figure 1D**). These findings were statistically confirmed with MANOVA (**Table 2**). These results suggested that RSA did alter the vaginal microbiome constituents.

**Table 2:** Results from nonparametric MANOVA analysis based on UniFrac distance.

Comparison	*p*-value (UniFrac distance)	
	Unweighted	Weighted
NM *vs.* Control	0.012	0.498
DT *vs.* Control	0.021	0.287
NM *vs.* DT	0.487	0.174
Metformin *vs.* Control	0.534	0.518
Aspirin plus Metformin *vs.* Control	0.078	0.270
Other medication *vs.* Control	0.033	0.467
Aspirin plus Metformin *vs.* Other medication	0.543	0.295
Aspirin plus Metformin *vs.* Metformin	0.540	0.540
Metformin *vs.* Other medication	0.349	0.866

NM, no medication; DT, drug treatment.

### Comparison of Vaginal Microbial Relative Abundance in Samples

**Figure 2:**
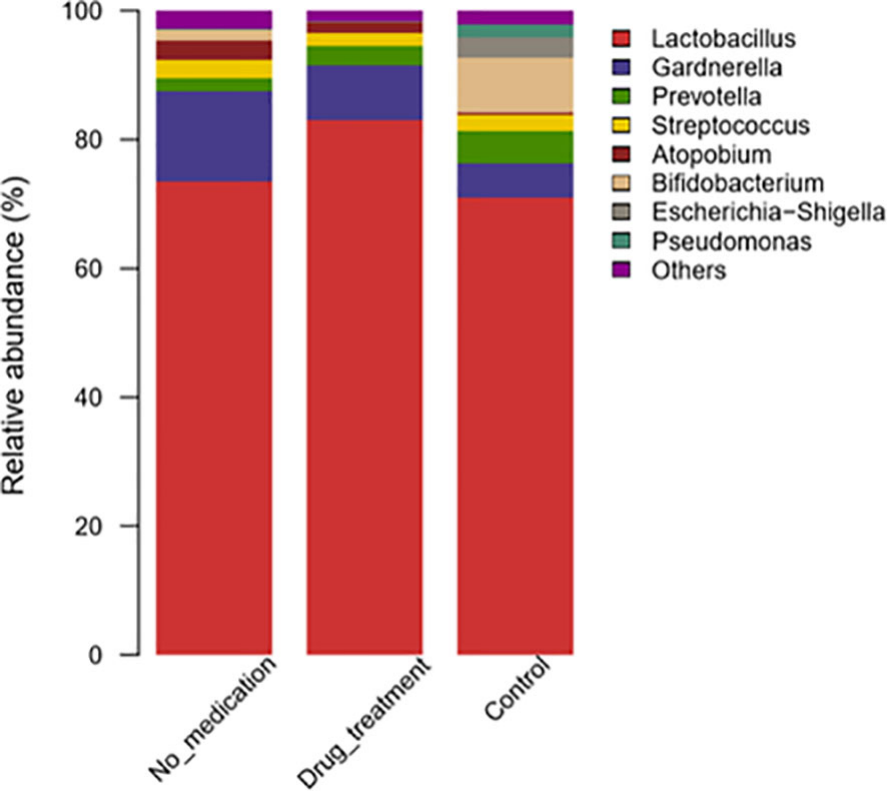
Taxonomic classification of the vaginal microbiota at the genus level among different groups.

At the genus level, differences in the abundance of vaginal microflora between cases and controls were evaluated by one-sided Wilcoxon rank-sum tests. Overall, at the genus level, the top eight dominant taxa were *Lactobacillus*, *Gardnerella*, *Prevotella*, *Streptococcus*, *Atopobium*, *Bifidobacterium*, *Escherichia/Shigella*, and *Pseudomonas* (**Figure 2**). In the no-medication group, *Lactobacillus* and *Gardnerella* were found in 73.52% and 14.01% subjects, respectively, and in the control group in 70.89% and 5.48% subjects, respectively. In patients receiving medication, the *Lactobacillus* genus constituted 82.92% of total bacteria and *Gardnerella*, 8.64%. *Prevotella* was present in low percentages in the no-medication (2.03%) and drug-treated groups (2.88%) compared to the controls (4.87%). In general, there was no significant difference in these top eight genera among groups.

*Megasphaera* and *Sneathia* were more abundant in the no-medication group than the control group (*p* = 0.013 and *p* = 0.051, respectively) (**Supplementary Table S1**). The relative abundances of *Lactobacillus* and *Sneathia* were high in the medication group compared to the control group (*p* = 0.051 and *p* = 0.071, respectively). After the subdivision of *Sneathia* species, it was found that the statistical difference between *Sneathia* in RSA group and the control group was caused by *Sneathia sanguinegens* (**Supplementary Table S2**).

**Figure 3:**
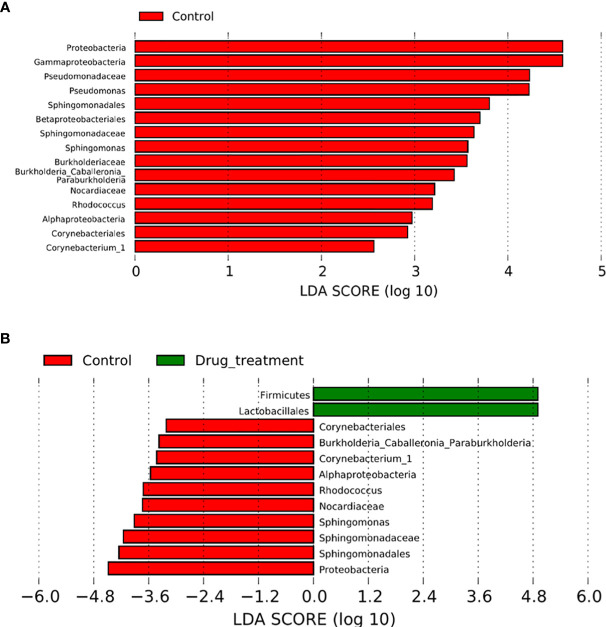
Comparison between patients with recurrent spontaneous abortion (RSA) and controls. The effect size for each differentially abundant taxon was computed using linear discriminant analysis (LDA), which indicated its contribution to group differentiation. OTUs are presented in red and green when the taxa were significantly more abundant in the controls and RSA, respectively. **(A)** Differentially abundant vaginal taxa detected in patients not taking any medicine and controls. **(B)** Differentially abundant taxa detected in patients who did take medicine and controls.

The relative abundances of 14 genera were significantly reduced in the no-medication group, and that of 23 genera were decreased in the medication group compared to controls (**Supplementary Tables S1, S3**). When the no-medication and medication groups were compared, nine genera with significantly different abundances were detected, of which eight were reduced in the medication group (**Supplementary Table S4**). LDA was used to identify the contribution of differentially abundant taxa to group differentiation. Five genera including *Corynebacterium_1*, *Rhodococcus*, *Burkholderia*_*Caballeronia*_*Paraburkholderia*, *Pseudomonas*, and *Sphingomonas* were significantly more abundant at the genus level in controls than in the no-medication group (**Figure 3A**). It is worth noting that after drug treatment, the relative abundance of *Lactobacillus* in patients was significantly higher than that in healthy women. In addition, when the patients were treated with medication, the relative abundance of *Pseudomonas* was the same as in controls (**Figures 3A, B** **)**. These results indicated that *Megasphaera, Sneathia sanguinegens, Corynebacterium_1, Rhodococcus*, *Burkholderia*-*Caballeronia*-*Paraburkholderia*, *Pseudomonas*, and *Sphingomonas* were strongly associated with RSA. This situation was altered to a certain extent after drug treatment.

### The Effect of Metformin Monotherapy or Combined With Aspirin on Vaginal Microbiota

**Figure 4:**
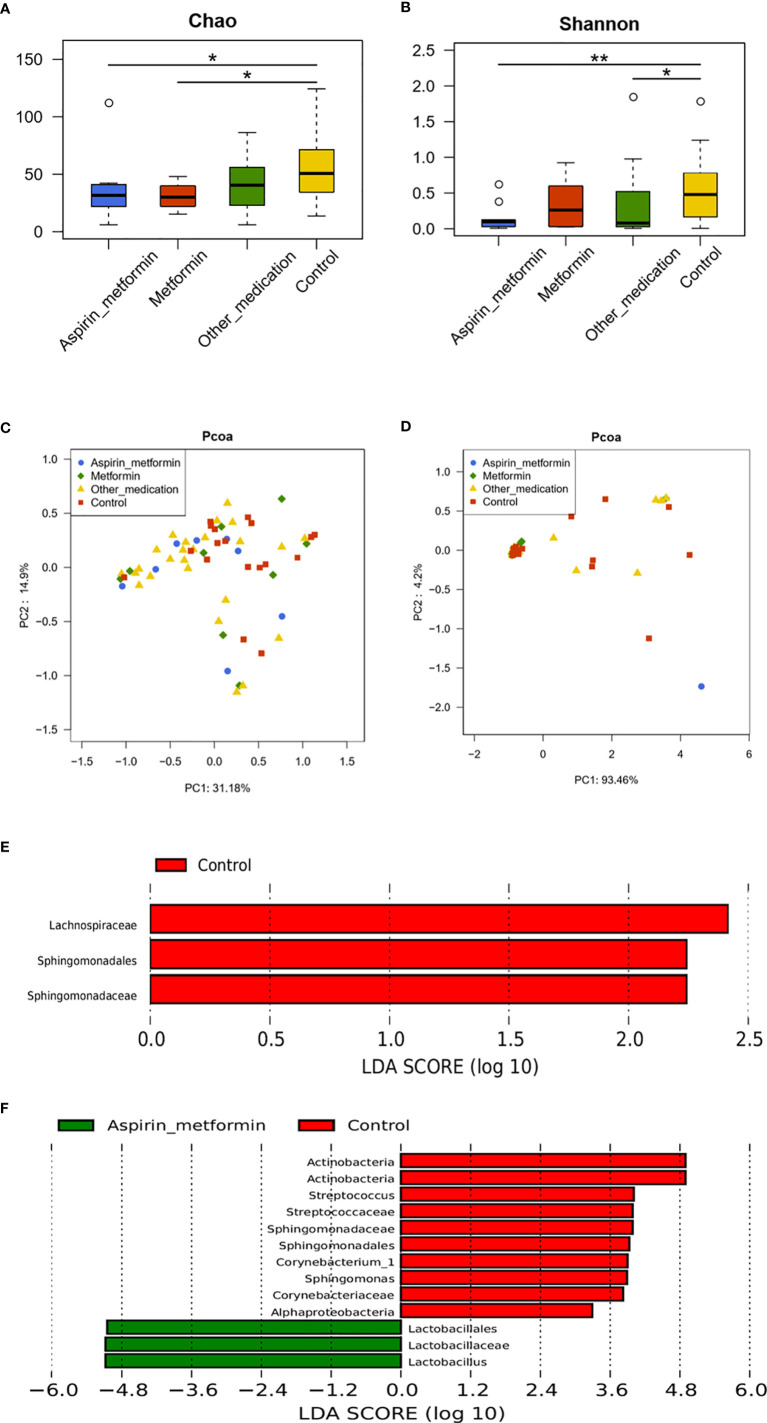
The effect of different medications on vaginal microbiota. The diversity of the vaginal microbiota in each group is shown by the Chao index **(A)**, Shannon–Wiener index **(B)**, and principal coordinate analysis (PCoA) plot created based on the unweighted **(C)** and weighted **(D)** UniFrac distances. **(E)** Differentially abundant vaginal taxa detected using LDA in samples taken from the metformin and control groups. **(F)** Differentially abundant taxa detected in patients who received metformin combined with aspirin and controls. **(A, B)** P values were calculated using the one-sided Wilcoxon rank-sum tests. *p < 0.05 and **p < 0.01.

Bacterial richness and diversity (**Figures 4A, B** **)** remained unchanged when patients were treated with metformin. When patients received aspirin combined with metformin, the diversity of the microbiota was decreased (**Figure 4B**). The microbiota profiles of the metformin and control groups clustered together after weighted and unweighted UniFrac analyses (**Figures 4C, D** **)**. These results were confirmed using the LEfSe (Linear Discriminant Analysis Effect Size) algorithm (**Figure 4E**). PCoA plots indicated that clustering of the metformin combined with aspirin group and control group could be separated to a certain degree by unweighted UniFrac analysis (*p* = 0.078) (**Figure 4C**). At the genus level, seven genera (*Lactobacillus*, *Aeromonas*, *Megasphaera*, *Bacteria*_*unclassified*, *Streptococcus*, *Sphingomonas, and Corynebacterium*) with significantly different relative abundances were detected in the metformin combined with aspirin group and control group (**Supplementary Table S5**). According to the LEfSe results, *Lactobacillus*, *Corynebacterium*_1, *Streptococcus*, and *Sphingomonas* played a key role regarding the differences between the two groups (**Figure 4F**). These findings showed that the composition of the patient’s vaginal microbiota tended to approach the vaginal microbial composition of the controls after metformin and aspirin exposure, and the abundances of *Lactobacillus* spp. were significantly increased after treatment with metformin plus aspirin.

### Characteristics of Vaginal Microbiome Community Structure Types (CSTs)

**Figure 5:**
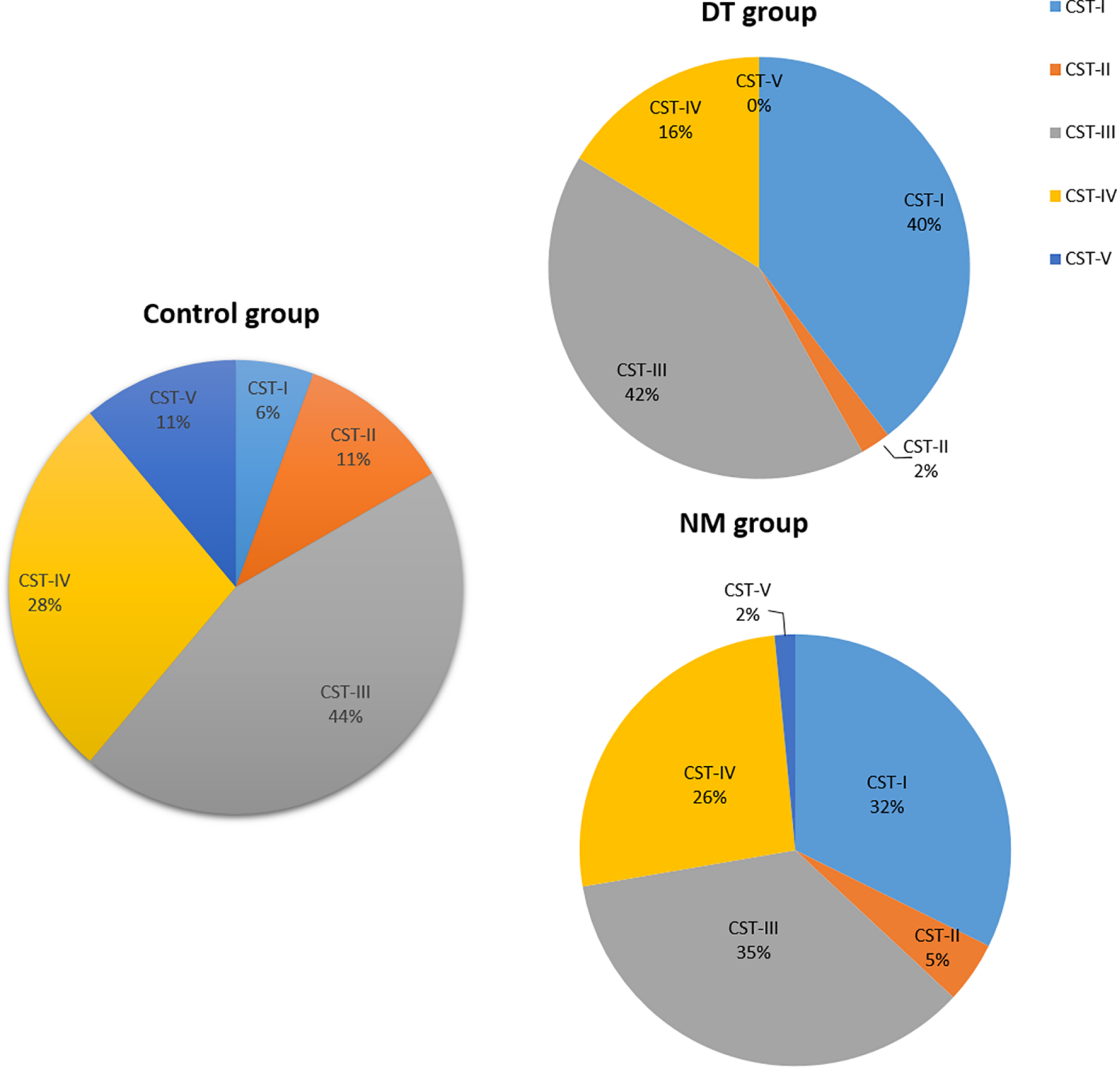
The proportion of the Community State Types (CSTs) in each group; NM group (*n* = 65), DT group (*n* = 43), control group (*n* =18).

Vaginal microbiota of 126 samples were identified as five vaginal CSTs (**Figure 5**). Overall, frequencies of CSTs in all samples were as follows: CST I, 30.95% (39/126), CST II, 4.76% (6/126), CST III, 38.89% (49/126), CST IV, 23.02%(29/126), and CSTV, 3/126 (2.38%). CST I was present in 32.31% (21/65) of the no-medication (NM) group, 39.53% (17/43) in the drug-treatment (DT) group and 5.56% in control group. CST I was present in 32.31% (21/65) in the NM group, 39.53% (17/43) in the DT group, and 5.56% in control group. The CST II cluster included 4.62%(3/65) of NM patients, 2.33% (1/43) of DT patients, and only 11.11% (2/18) of the heathy controls. CST III accounted for 35.38% (23/65) of the NM group, 41.86% (18/43) in the DT group, and 44.44% (8/18) in the control group. CST IV included 26.15% (17/65) of the NM group, 16.28% (7/43) in the DT group, and 27.78%(5/18) in the control group. Only one case of CSTV was found in the NM group, none in DT group, and two cases in control group. In the NM group, only one case of CSTV was found, accounting for 1.53% (1/63); this type was not found in the DT group. Two cases were found in the controls, accounting for 11.11% (2/18). There was no statistical difference in the distribution of CST between the NM group and the NT group. Comparing the CST distribution of the control group with the NM and DT groups respectively, there was significant statistical significance (*p* = 0.042 and *p* = 0.006, respectively), with a statistically significant increase of CST I in NM and DT patients compared to the control group (5.56% *vs.* 32.31%, *p* < 0.05; 5.56% *vs.* 39.53%, *p* < 0.05).

## Discussion

The pathogenesis of RSA is complex, and at least half of the cases do not seem to have any concrete underlying cause. The use of next-generation sequencing technology to determine the characteristics of vaginal microbes in patients with RSA may provide new clues for exploring the etiology. A comprehensive understanding of the characteristics of vaginal microbiota in RSA and its response to drug treatment is essential to improve diagnosis and treatment strategies. Our study showed that patients with RSA have decreased richness of the vaginal microbiome, along with unchanged dominant *Lactobacillus* spp. and increased abundances of *Megasphaera* and *Sneathia sanguinegens*. Additionally, the vaginal microecology composition in the patients was different from that of healthy women. The difference was attenuated to a certain extent after drug treatment. Treatment with metformin combined with aspirin might significantly increase the abundance of vaginal *Lactobacillus* spp. in patients.

According to previous reports, factors including age, BMI, hygiene habits, diet, exercise intensity, smoking, and perceived stress can affect the vaginal microbiome (ESHRE Guideline Group on RPL et al., 2018; He et al., 2019; Khalife et al., 2019; Turpin et al., 2019; Song et al., 2020). In this study, all factors except for age were similar among the experimental groups (**Table 1**). This is likely because healthy women in the control group had successfully conceived twice and were older than those in the case group. Based on the results of statistical analysis, the difference in age between the experimental groups has no effect on the comparison of differences in microbiota between groups.

As the dominant organism of the vaginal community, *Lactobacilli* play a key role in maintaining vaginal health. *Lactobacillus* species inhibit the growth of other microorganisms by producing lactic acid through metabolizing extracellular glycogen to lower the pH of the vaginal environment, producing bacteriocins and hydrogen peroxide (H_2_O_2_), and competing for nutrients and space, thereby protecting the vaginal ecosystem from harmful microbial communities. (Aroutcheva et al., 2001; Caselli et al., 2020). Therefore, vaginal pH (greater than 4.5) is one of the important indicators for the diagnosis of bacterial vaginosis in clinical practice (Verstraelen and Verhelst, 2009). H_2_O_2_ is generally considered to prevent bacteria associated with bacterial vaginosis (Eschenbach et al., 1989; Beigi et al., 2005). However, the ability of different types of *Lactobacillus* to produce organic acids, bacteriocins, and H_2_O_2_ is not completely the same (Eschenbach et al., 1989; Aroutcheva et al., 2001). Herein, there was no statistical difference in the measurement of vaginal pH and H_2_O_2_ in all participants between each group. This might be due to the similar distribution of vaginal *Lactobacillus* species, which can produce organic acids and H_2_O_2_ in each group. Sialidases are enzymes widely present in bacteria, fungi, viruses, mycoplasma, and animals that catalyze the removal of sialic acid from various glycoconjugates (Taylor, 1996). Many of these pathogens use sialidase to assist their pathogenesis and/or nutritional requirements. The detection of vaginal sialidase activity has also been used in the auxiliary diagnosis of bacterial vaginosis (Myziuk et al., 2003; Paladine and Desai, 2018). In the present study, vaginal sialidase activity was negative in all subjects. LE, an indicator of inflammatory cells present in tissues or samples, is used to evaluate urogenital tract infections and periprosthetic joint infection (Abbasi et al., 1985; Mårdh et al., 2003; Lee et al., 2017). It was found that LE activity of RSA group was higher than that of the control group, and the difference was statistically significant. No significant difference was found between no-medication group and drug treatment group in the test of LE activity. LE tests were weakly positive in 46.2% of patients with RSA. In the control group, there was only one positive case of LE test, accounting for 5.5%, and no weak positive case was found. This finding suggested that nearly half of the patients in case group may have mild inflammation in the vagina. The reason of this mild inflammation might have to do with changes in their vaginal microbiome composition.

Recent studies have found that the diversity and richness of vaginal microbiota in women who miscarried in the first and second trimesters were significantly higher than those of normal controls (Al-Memar et al., 2019). Our results showed that compared with healthy women, non-pregnant patients with RSA have reduced vaginal microbiome richness and unchanged vaginal flora diversity. In contrast, Zhang et al. showed that there was no significant difference in flora richness and diversity compared with the control group (Zhang et al., 2019). This could be attributed to the significant difference in the number of patients in the two articles, and the fact that their study focused on 10 patients with unexplained miscarriage. Flora diversity was decreased when patients received aspirin combined with metformin treatment or other medications including immunomodulators, low-dose steroids, and/or Chinese medicine, except metformin.

At the genus level, predominant microbiota, including *Lactobacillus*, *Gardnerella*, *Prevotella*, *Streptococcus*, *Atopobium*, *Bifidobacterium*, *Escherichia/Shigella*, and *Pseudomonas* were not significantly different between the no-medication patient and control groups. However, Zhang et al. reported that *Atopobium*, *Prevotella*, and *Streptococcus* were more abundant in 10 patients and *Lactobacillus* and *Gardnerella* were overrepresented in 10 controls (Zhang et al., 2019). Kuon et al. indicated that patients with RSA had more colonization by *Gardnerella vaginalis* and Gram-negative anaerobes including *Prevotella*, *Bacteroides*, and *Veillonella* species, based on culture-dependent methods (Kuon et al., 2017). We speculate that the reason for the inconsistent conclusions of these two studies is because of differences in sample size and research methods.

According to the prevalence of *Lactobacillus* and other bacteria in the vaginal flora, CSTs are divided into five types. Among these five CSTs, four of them are predominated by species of *Lactobacillus* (CST I, *L. crispatus*; CST II, *L. gasseri*; CST III, *L. iners*; and CST V, *L. jensenii*). CST IV is composed of diverse bacteria dominated by anaerobic bacteria (Ravel et al., 2011). According to the analysis of the vaginal microbiome characterization of all subjects, it was found that there were statistical differences in the classification of the vaginal flora between the control group and the case group including no-medication and drug-treatment groups. No significant difference was found between no-medication patients and drug-treatment patients in the frequencies of CSTs. This may be caused by a different vaginal microbiota structure in patients than in controls. It is in line with the conclusion that RSA is associated with the changes in vaginal microbiome constituents as analyzed by PCoA. The prevalence of each CST within the three groups was compared and no statistical differences was found except in CST I. The prevalence of CST I was higher in the patient group than in the control group. The protective role of *Lactobacillus s*pecies, including *L. crispatus*, was demonstrated in many previous studies. (Aroutcheva et al., 2001; Hočevar et al., 2019; Caselli et al., 2020). This difference may be attributed to the small sample size of control subjects and the effect of drug therapy on patients’ vaginal microbiome profile. Prevalence of CST IV in the first trimester stages was correlated with gestational age at delivery (DiGiulio et al., 2015). By contrast, Elovitz et al. suggested that the prevalence of *Lactobacillus* spp. was not associated with spontaneous preterm birth after conducting a study involving a prospective cohort of 2,000 single pregnancy women (Elovitz et al., 2019). In future, a larger sample size is needed to analyze the characteristics of vaginal CSTs between patients with RSA and healthy controls.

The abundances of 15 genera were significantly different between the no-medication and control groups (*p* < 0.05). However, in untreated patients, *Megasphaera* abundance was significantly increased. The abundance of *Sneathia sanguinegens* in untreated patients was also higher than in controls, and this difference showed a trend toward statistical significance (*p* = 0.051). Therefore, *Sneathia sanguinegens*, *Megasphaera*, and the reduced 14 genera might be related to RSA. *Megasphaera* was also previously shown to have a positive association with PTB in studies by of Hočevar et al. and Nelson et al. (Nelson et al., 2014; Hočevar et al., 2019). *Sneathia* spp., which can adhere to cervical epithelial cells and have a high cytotoxic potential, were previously associated with serious pregnancy complications including spontaneous abortions, preterm labor, and preeclampsia resulting from invasion into the uterine cavity and amniotic sac (Harwich et al., 2012; Seo et al., 2017; Fettweis et al., 2019). Gentile and colleagues recently discovered a novel virulence-related CptA produced by *S. amnii* that can permeabilize chorionic trophoblast cells and lyse human red blood cells (Gentile et al., 2020). Our findings showed that *Sneathia sanguinegens* might have a specific role in RSA. Of the 14 reduced bacterial genera, 5 (*Corynebacterium_1*, *Rhodococcus*, *Sphingomonas*, *Burkholderia-Caballeronia-Paraburkholderia*, and *Pseudomonas*) contributed the most to the difference between controls and cases on in-depth analysis by LDA, meaning that they were most closely related to RSA. All five genera are composed of aerobic bacteria, and further studies are needed to explore the underlying mechanism of this phenomenon.

Metformin is an effective antidiabetic drug that is also used to treat endocrine diseases caused by PCOS (e.g., polycystic ovary-related recurrent miscarriage) and normalize endocrine, metabolic, and reproductive functions (Rai and Regan, 2006; Practice Committee of the American Society for Reproductive Medicine, 2012; Khalife et al., 2019; Wu et al., 2020). Metformin has attracted much attention in recent years because of its anticancer effects on several human solid tumors (Yu et al., 2019). Some scholars speculated that the benefits of metformin in cancer prevention and treatment might be mediated by the intestinal flora (Wu et al., 2020). Given its antiplatelet and anti-inflammatory properties, aspirin is often used to prevent and treat cardiovascular diseases, as well as for the treatment of unexplained recurrent miscarriage and autoimmune-related recurrent miscarriage. Aspirin has also been proven to have antitumor effects, especially in colorectal cancer; this might be due to the relatively favorable migration of the intestinal microbiome caused by aspirin (Wu et al., 2020). In our study, the vaginal microbiota composition of patients significantly recovered when they received metformin alone. After administration of metformin plus aspirin, patients’ vaginal microbial composition was partially restored. Specifically, at the genus level, the difference between the cases and controls was reduced from the original difference of 15 genera to 6 genera. It is worth noting that when patients were treated with metformin and aspirin, the abundance of vaginal *Lactobacillus* spp. increased significantly. To our knowledge, this has not been reported in past studies and provides new ideas for the future treatment of diseases caused by the decrease or absence of *Lactobacillus spp*. Ideally, further in-depth research studies should be performed on larger cohorts of subjects with RSA to verify our results and explore related mechanisms.

## Conclusion

Patients experiencing RSA presented a less rich vaginal microbiome with decreased abundance of *Pseudomonas*, *Burkholderia-Caballeronia-Paraburkholderia*, *Corynebacterium_1*, *Rhodococcus*, and *Sphingomonas*, along with increased abundance of *Megasphaera* and *Sneathia sanguinegens*. Drug treatment affects vaginal microbiota composition. Metformin alone or in combination with aspirin might normalize microbiota composition. Metformin plus aspirin might significantly increase the abundance of vaginal *Lactobacillus* spp.

## Data Availability Statement

The datasets presented in this study can be found in online repositories. The names of the repository/repositories and accession number(s) can be found below: NCBI SRA; PRJNA683172.

## Ethics Statement

The studies involving human participants were reviewed and approved by the Ethics Committee of the Obstetrics and Gynecology Hospital of Fudan University. The patients/participants provided their written informed consent to participate in this study.

## Author Contributions

CY, MD, and FZ conceived and designed the study. JG and ZW contributed to obtaining ethical approval. YC was responsible for financial management. Participant recruitment and sample collection were conducted by MD and FZ. Experiments and data collection were performed by ZW, CL, YC, NH, and FZ. Data were analyzed by JG, MW, MD, FZ, CY, and LC. Figures and tables were generated by FZ and LC. The manuscript was written by FZ and reviewed by CY and MD. All authors contributed to the article and approved the submitted version.

## Funding

Financial support was provided by the National Nature Science Foundation of China (grant number: 81873970). 

## Conflict of Interest

NH and LC were employed by the company Sinotech Genome Technology Co., Ltd.

The remaining authors declare that the research was conducted in the absence of any commercial or financial relationships that could be construed as a potential conflict of interest.

## Publisher’s Note

All claims expressed in this article are solely those of the authors and do not necessarily represent those of their affiliated organizations, or those of the publisher, the editors and the reviewers. Any product that may be evaluated in this article, or claim that may be made by its manufacturer, is not guaranteed or endorsed by the publisher.
