# Supplementary material for: Characterization of Vaginal Microbiota in Women With Recurrent Spontaneous Abortion That Can Be Modified by Drug Treatment
Source: Front Cell Infect Microbiol. 2021 Aug 19;11:680643. doi: 10.3389/fcimb.2021.680643 (PMC8417370; doi:10.3389/fcimb.2021.680643)
Supplement: Supplementary file 1 [file DataSheet_1.pdf]

Supplementary Table 1. Comparison of the relative abundances at the genus level between the no-medication and control groups.

Genus	NM group (n=65) Relative abundance (%)	Control group (n=18) Relative abundance (%)	P-value
Megasphaera	0.835±2.803	0±0	0.013
Sneathia	0.572±2.144	0±0	0.051
Gemella	0.017±0.062	0±0	0.078
Rhodococcus	0.050±0.165	0.137±0.311	0.012
Burkholderia−Caballeronia−Paraburkholderia	0.005±0.014	0.285±1.164	0.000
Pseudomonas	0.022±0.110	1.961±8.312	0.032
Bacteria_unclassified	0.015±0.033	0.038±0.054	0.007
Sphingomonas	0.012±0.056	0.014±0.023	0.003
Corynebacterium_1	0.003±0.004	0.002±0.008	0.038
Staphylococcus	0.001±0.003	0.049±0.193	0.029
Sediminibacterium	0±0.002	0.004±0.009	0.001
Pelomonas	0±0.000	0.007±0.022	0.049
Cutibacterium	0±0	0.002±0.005	0.016
0319−6G20_norank	0±0	0.001±0.004	0.031
BD7−11_norank	0±0	0.002±0.006	0.001
Obscuribacterales_norank	0±0	0±0.002	0.007
Sulfuritalea	0±0	0±0.002	0.009

NM, no medication.
