# Supplementary material for: Characterization of Vaginal Microbiota in Women With Recurrent Spontaneous Abortion That Can Be Modified by Drug Treatment
Source: Front Cell Infect Microbiol. 2021 Aug 19;11:680643. doi: 10.3389/fcimb.2021.680643 (PMC8417370; doi:10.3389/fcimb.2021.680643)
Supplement: Supplementary file 2 [file DataSheet_2.pdf]

Supplementary Table 2. Comparison of the relative abundances of *Sneathia* species between the no-medication, drug-treatment and control groups.

Genus	NM group (n=65) Relative abundance (%)	DT group (n=43) Relative abundance (%)	Control group(n=18) Relative abundance (%)	P value		
				NM vs. DT	NM vs. Control	DT vs. Control
Sneathia sanguinegens	0.564±2.115	0.396±2.044	0±0	0.674	0.051	0.072
Sneathia amnii	0.009± 0.046	0.011± 0.051	0±0	0.970	0.184	0.185

NM, no medication; DT, drug treatment.
