# Supplementary material for: Characterization of Vaginal Microbiota in Women With Recurrent Spontaneous Abortion That Can Be Modified by Drug Treatment
Source: Front Cell Infect Microbiol. 2021 Aug 19;11:680643. doi: 10.3389/fcimb.2021.680643 (PMC8417370; doi:10.3389/fcimb.2021.680643)
Supplement: Supplementary file 3 [file DataSheet_3.pdf]

Supplementary Table 3. Comparison of the relative abundance at the genus level between the drug-treatment and control groups.

Genus	DT group (n=43) Relative abundance (%)	Control group (n=18) Relative abundance (%)	P-value
Lactobacillus	82.924±35.794	70.896±38.941	0.051
Rhodococcus	0.030±0.069	0.137±0.311	0.028
Burkholderia−Caballeronia−Paraburkholderia	0.003±0.005	0.285±1.164	0.001
Bacteria_unclassified	0.011±0.022	0.038±0.054	0.003
Staphylococcus	0.000±0.001	0.049±0.193	0.005
Pelomonas	0.000±0.000	0.006±0.022	0.006
Sphingomonas	0.005±0.020	0.014±0.023	0.001
Chitinophagaceae_uncultured	0.000±0.000	0.004±0.013	0.008
Sediminibacterium	0.000±0.000	0.003±0.008	0.001
Corynebacterium_1	0.000±0.002	0.003±0.004	0.024
env.OPS_17_norank	0.000±0.000	0.002±0.008	0.009
Bradyrhizobium	0.000±0.000	0.002±0.008	0.034
Cutibacterium	0±0	0.002±0.004	0.000
BD7-11_norank	0±0	0.002±0.005	0.001
Corynebacterium	0.000±0.000	0.001±0.005	0.038
0319-6G20_norank	0±0	0.001±0.004	0.006
Mycoplasma	0±0	0.001±0.004	0.027
Bacteroides	0.000±0.000	0.001±0.002	0.034
Bdellovibrio	0±0	0.000±0.003	0.027
Obscuribacterales_norank	0.000±0.000	0.000±0.002	0.027
Blautia	0±0	0.000±0.001	0.027
Parvimonas	0.000±0.000	0.000±0.001	0.027
Negativicoccus	0±0	0.000±0.001	0.027
Rhodocyclaceae_unclassified	0±0	0.000±0.001	0.027

DT, drug treatment.
