# Supplementary material for: Characterization of Vaginal Microbiota in Women With Recurrent Spontaneous Abortion That Can Be Modified by Drug Treatment
Source: Front Cell Infect Microbiol. 2021 Aug 19;11:680643. doi: 10.3389/fcimb.2021.680643 (PMC8417370; doi:10.3389/fcimb.2021.680643)
Supplement: Supplementary file 4 [file DataSheet_4.pdf]

Supplementary Table 4. Relative abundance of genera discovered in samples that were significantly different between the no-medication and drug treatment groups at the genus level.

Genus	NM group (n=65) Relative abundance (%)	DT group (n=43) Relative abundance (%)	P-value
Megasphaera	0.835±2.804	0.386±2.391	0.042
DNF00809	0.199±0.945	0.001± 0.004	0.021
Parvimonas	0.052±0.210	0±0	0.004
Gemella	0.017±0.062	0±0.001	0.026
Mycoplasma	0.002± 0.008	0±0	0.017
Actinobacteria_unclassified	0.002± 0.008	0±0	0.041
Mobiluncus	0.002± 0.005	0±0.005	0.047
Blautia	0±0.001	0±0	0.041
Cloacibacterium	0±0.001	0±0	0.031

NM, no medication; DT, drug treatment.
