# Supplementary material for: Characterization of Vaginal Microbiota in Women With Recurrent Spontaneous Abortion That Can Be Modified by Drug Treatment
Source: Front Cell Infect Microbiol. 2021 Aug 19;11:680643. doi: 10.3389/fcimb.2021.680643 (PMC8417370; doi:10.3389/fcimb.2021.680643)
Supplement: Supplementary file 5 [file DataSheet_5.pdf]

Supplementary Table 5. Relative abundance of genera discovered in the samples that were significantly different between the metformin plus aspirin and control groups.

Genus	Metformin combined with aspirin group (n=9) Relative abundance (%)	Control group (n=18) Relative abundance (%)	P-value
Lactobacillus	88.610±33.229	70.896±38.941	0.042
Aeromonas	0.003± 0.006	0	0.010
Megasphaera	0.002± 0.005	0	0.041
Bacteria_unclassified	0.003±0.004	0.038±0.054	0.009
Streptococcus	0±0	2.499±0.026	0.013
Sphingomonas	0.000±0.000	0.014±0.023	0.003
Corynebacterium	0.000±0.000	0.003±0.004	0.047
