# Supplementary material for: Characterization of Vaginal Microbiota in Women With Recurrent Spontaneous Abortion That Can Be Modified by Drug Treatment
Source: Front Cell Infect Microbiol. 2021 Aug 19;11:680643. doi: 10.3389/fcimb.2021.680643 (PMC8417370; doi:10.3389/fcimb.2021.680643)
Supplement: Supplementary file 6 [file DataSheet_6.pdf]

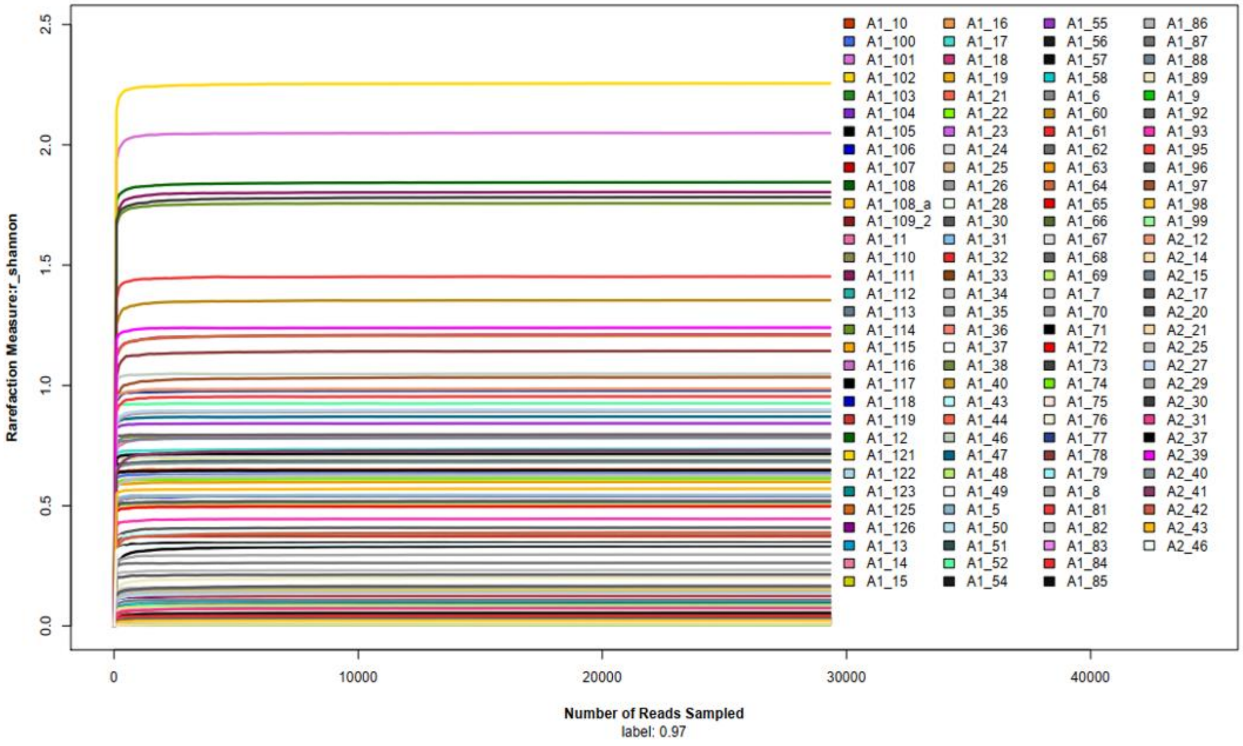

Figure S1 Shannon-Wiener curves of all samples. The horizontal axis represents the number of reads sampled, and the vertical axis represents the Shannon index. Each color represents a sample.
